# Assessment of Demographic and Socio-Behavioral Factors on Adherence to HIV Pre-Exposure Prophylaxis Using a Markov Modeling Approach

**DOI:** 10.3389/fphar.2019.00785

**Published:** 2019-07-12

**Authors:** Surulivelrajan Mallayasamy, Ayyappa Chaturvedula, Michael J. Fossler, Mark E. Sale, Craig W. Hendrix, Jessica E. Haberer

**Affiliations:** ^1^UNT System College of Pharmacy, UNTHSC, Fort Worth, TX, United States; ^2^Trevena Inc, King of Prussia, PA, United States; ^3^Nuventra, Raleigh, NC, United States; ^4^Johns Hopkins University School of Medicine, Baltimore, MD, United States; ^5^Massachusetts General Hospital and Harvard Medical School, Boston, MA, United States

**Keywords:** adherence, Markov model, HIV, preexposure prophylaxis, covariates

## Abstract

**Purpose:** Adherence is important for the effectiveness of human immunodeficiency virus (HIV) preexposure prophylaxis (PrEP). The objective of the current work is to assess the impact of multiple demographic and socio-behavioral factors on the adherence to tenofovir-based PrEP among HIV serodiscordant couples in East Africa using Markov mixed-effects modeling approach.

**Methods:** The Partners Demonstration Project was a prospective, open-label, implementation science-driven study of HIV PrEP among heterosexual HIV serodiscordant couples in Kenya and Uganda. The uninfected partner received oral PrEP according to the “bridge to antiretroviral therapy [ART]” strategy (i.e., until the infected partner had been on ART for ≥6 months). Adherence was monitored electronically; demographic and socio-behavioral data were collected during study visits. Analyzed data reflect 12 months of follow-up per participant. A two-state, first-order, discrete time Markov model was developed with longitudinal adherence data characterized by “dose taking (1)” and “dose missing (0).” Covariate effects were linearly added in the logit domain of transition probability parameters (P01 and P10) in the model. The full covariate model was initially developed, followed by backward elimination process to reduce the model. All significant covariates reported by a prior primary statistical analysis of the same data were included in the full covariate model.

**Results:** The model included data from 920 participants, who were predominantly male (65%). Significant covariates associated with higher adherence were 25 years or older [odds ratio (OR) for P10, 0.61], female sex (OR for P10, 0.67), participant wanting the relationship with the partner to succeed (OR for P10, 0.79; OR for P01, 1.45), and sex with partner either with 100% or <100% condom use compared to those reported no sex (OR for P10, 0.84; OR for P01, 1.21). Significant covariates associated with lower adherence were partner on ART >6 months (OR for P01, 0.86; OR for P10, 1.34), subject in the study for >6 months (OR for P01, 0.8; OR for P10, 1.25), and problematic alcohol use (OR for P01, 0.63; OR for P10, 1.16).

**Conclusion:** The developed Markov model provides a mechanistic understanding of relationship between demographic, socio-behavioral covariates, and PrEP adherence, by indicating the pattern of adherence influenced by each factor over time. Such data can be used for further intervention development to promote PrEP adherence.

## Introduction

The oral formulation of tenofovir disoproxil fumarate (TDF) in a fixed-dose combination with emtricitabine (FTC) was approved by the U.S. FDA in 2012 for preexposure prophylaxis (PrEP) to reduce the risk of sexually acquired HIV-1 infections. Adherence is highly correlated to the prophylactic efficacy in various clinical trials (Haberer, 2016). Adherence is defined as the extent to which a person’s behavior corresponds with medications, diet, and lifestyle as recommended by a health care provider (World Health Organization, 2003). Adherence involves three distinct components known as initiation, execution, and persistence of prescribed therapy (Blaschke et al., 2012; Vrijens and Urquhart, 2014), and non-adherence can be seen in any one or all of these components (Vrijens et al., 2012). Non-adherence to medications is complex and may be influenced by various domains, including socio-economic, health system, disease condition, treatment, or patient-related factors. Understanding factors associated with these domains is the key to understanding adherence-related problems in a holistic manner and designing interventions to suitably address them (World Health Organization, 2003).

Monitoring adherence in clinical trials and routine patient care is a difficult task that requires resources and staffing. Direct and indirect monitoring methods have been employed to monitor adherence; each method has its own advantages and shortcomings (Farmer 1999; Lam and Fresco, 2015). Directly observed therapy (DOT), which verifies adherence, is the most reliable method and has been a mainstay in many tuberculosis treatment protocols, but is highly resource-intensive (Chaulk and Kazandjian, 1998). Self-report by patients and pharmacy refill are easy-to-implement indirect methods, but they tend to overestimate adherence (El Alili et al., 2016). Electronic monitoring methods, such as the Medication Event Monitoring System (MEMS^®^), involve containers that record each opening as a proxy for medication-taking behavior; this approach has been found to be more reliable than patient self-reports and has been used in numerous clinical trials (Van Onzenoort et al., 2010;Riekert and Rand, 2002). Pharmacological measures of treatment adherence in PrEP are gaining prominence in clinical practice (Brooks and Anderson, 2018; Hendrix, 2018). TDF is a prodrug that rapidly hydrolyzed to tenofovir (TFV) in plasma and further phosphorylated to an active intracellular metabolite, TFV diphosphate (TFV-DP). Establishing adherence benchmarks of TFV and TFV-DP concentrations for HIV PrEP was recently conducted through a DOT study design (Hendrix et al., 2016). This approach has a limited view into past dosing information and does not provide high resolution of patterns of adherence. Importantly, electronic monitoring is the only method that provides day-to-day records of longitudinal data, thus providing an opportunity to understand the adherence patterns of the population in a detailed manner (Osterberg and Blaschke, 2005).

Adherence is commonly expressed as a summary measure, such as percentage adherence, which does not take into account variations in adherence patterns over time (Brown and Bussell, 2011). Non-therapeutic time (NTT) is an alternative to summary measures of adherence and may be calculated using electronic adherence data; it is expressed as a sum of all non-therapeutic intervals during a course of therapy and reflects cumulative non-adherence (Girard et al., 1998).

Electronic adherence monitoring provides longitudinal data on dose-taking behavior. Adherence can be described as sequence of two discrete states, such as “dose-taking” and “dose-missing.” Because prior states may influence future states, analytic methods should account for within subject correlation. In addition to the within subject correlation, there could be a dependence between the successive outcomes. If the future evolution of a system depends only on the current state, but not on the history, then the system can be considered to exhibit the Markov property (Stewart, 2009). It has been shown that ignoring Markovian tendencies in the data could lead to elevated type 1 error rates in covariate selection (Silber et al., 2009). Assessing transitions between or within the two states is the basis for a Markov modeling approach. Adherence can be quantified and modeled by deriving probability parameters for transition between the discrete states that explain an individual’s dose-taking pattern over a period. A Markov mixed-effects modeling approach has been implemented for analyzing adherence, describing drug holiday patterns, and identifying influential covariates of PrEP adherence from the MEMS-documented adherence data sets (Girard et al., 1998; Fellows et al., 2015; Madrasi et al., 2017). The Markov modeling approach has also been used to analyze data in diverse studies, such as those dealing with adverse effects of drugs, seizures counts, and patterns of sleep stages (Karlsson et al., 2000; Zingmark et al., 2005; Ito et al., 2008; Henin et al., 2009; Bisaso et al., 2015).

Adherence is particularly important for the effectiveness of HIV PrEP (Haberer, 2016). With high adherence, TDF and emtricitabine (FTC) in combination have been successfully used as a PrEP regimen in high-risk groups. A number of demonstration projects are currently underway globally using this regimen (Global Advocacy for HIV prevention, 2017). Understanding the covariates that influence adherence patterns is vital in designing appropriate patient counselling and interventions that will lead to better adherence. The Partners Demonstration Project involved delivery of TDF-FTC as PrEP to the HIV-uninfected members of heterosexual HIV serodiscordant couples in East Africa. PrEP adherence was measured electronically. The objective of this analysis was to assess the impact of demographic, social, and behavioral attributes on PrEP adherence using Markov mixed-effects modeling approach.

## Methods

### Study Participants and Enrollment

The Partners Demonstration Project was a prospective, open-label, implementation science-driven study of HIV PrEP among heterosexual HIV serodiscordant couples in Kenya and Uganda. Ethical statements, subject enrollment, and follow-up details are described in the primary publication on this study (Baeten et al., 2016). Briefly, serodiscordant couples were enrolled into the study, and the HIV-uninfected partner of the couple was encouraged to take PrEP (combination of emtricitabine 200 mg/TFV disoproxil fumarate 300 mg once daily) until the partner living with HIV had been on antiretroviral therapy (ART) for at least 6 months, when viral suppression was assumed (the “bridge” strategy), and if there were no concerns about ART adherence and/or the HIV status of additional partners. Medication was provided in a MEMS container (AARDEX Group, Switzerland). Study participants’ adherence records were downloaded from the containers during their follow-up study visits (1 month after enrollment, then quarterly for up to 2 years).

Demographic and socio-behavioral data were collected during study visits as well; details on the measures used are published in the primary analysis of these data (Haberer et al., 2017a).

### Data Set Preparation

Electronic adherence data consisted of the dates and times of the medication container openings, which are considered as a surrogate for dose-taking events. Multiple openings in a day were considered as only one dose-taking event on that day. Since the active metabolite, TFV-DP, has a long half-life (48 to 125 h) (Duwal et al., 2012; Louissaint et al., 2013), dose-time errors in a single day were not expected to significantly impact the therapeutic efficacy. Covariates based on the results of the primary analysis of this study were incorporated into the data set (Haberer et al., 2017a).

### Analysis of Adherence Transition States

The transition between dose-missing and dose-taking events in each of the two subsequent, adjacent days was assessed for the adherence data. Four transitions are possible in the data: [01], [11], [10], and [00], as per the first-order Markov model. For generating this transition for the first day in an individual, the previous state was considered as 1, which signifies a “dose taken” state. The first dosing state was assumed to be 1 based on the high overall adherence; moreover, prior modeling showed limited impact of the initial state assumption on outcomes (Madrasi et al., 2017). Transitions [01] and [11] signify transition from a “dose-missing” state to a “dose-taken state” and staying in a “dose-taking” state, respectively. Transitions [10] and [00] signify transition from a “dose-taking” state to a “dose-missing” state and continuing in a “dose-missing” state, respectively. The total number of each of these four transitions was calculated for each covariate.

### Model Development

Electronic adherence data available for the entire duration of observation (up to 24 months of follow-up) was used for base model selection. To match the primary statistical analysis (Haberer et al., 2017a), covariate analysis was conducted on the data until 12 months of follow-up. Adherence data were modeled using a logistic model, as well as a Markov model to identify the suitable approach (base model) to describe the data. The logistic model assumes adherence as series of coin flips and this model can be described as follows:

(1) $$\text{LOGIT}=\mathrm{Ln}\frac{P1}{1-P1}=\Theta_{1}+\eta i$$

where Θ_1_ is the population parameter of probability in the logit domain and η*i* is the between subject variability with a mean of zero and variance of ω^2^.

The probability (P1) of dose-taking was modeled in the logit domain and then transformed back to the probability space (between 0 and 1) as follows:

(2) $$P1=\left(\frac{e^{\Theta 1}}{1+e^{\Theta 1}}\right)$$

The probability of missing dose (P0) is defined as follows:

(3) P0=1–P1

**Figure 1:**
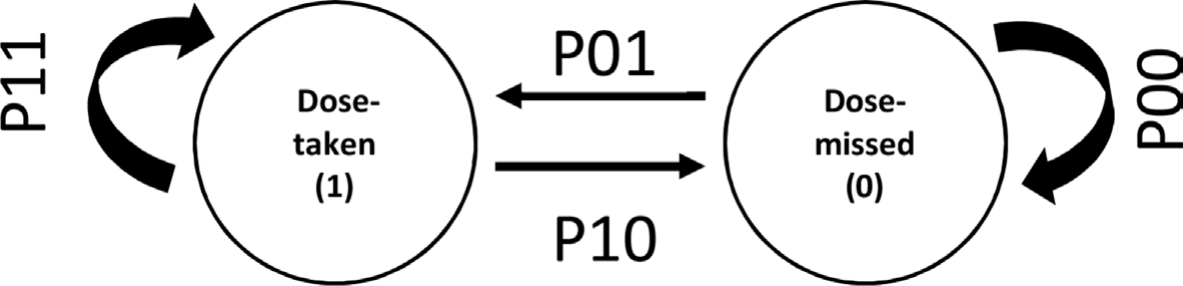
Discrete states and transitions representing adherence behavior in a Markov model. P01 and P10 represent transition probabilities between two states of adherence viz. “dose taken” and “dose missed.” P11 and P00 represent the transition probabilities for continuing same states.

The Markov model describes adherence as a series of transitions from the dose-taking state and dose-missing state defined by transition probabilities (**Figure 1**). The probabilities of transition between states were parameterized using logit functions. The model can be described as follows:

(4) $$\text{LOGIT}1=Ln\frac{P01}{1-P01}=\Theta_{1}+\eta i$$

(5) $$\text{LOGIT}2=Ln\frac{P10}{1-P10}=\Theta_{2}+\eta i$$

where Θ1 and Θ2 are the population parameters of the transition probability in the logit domain, and η*i* is the between-subject variability with a mean of zero and variance of ω^2^.

The transition probabilities between the discrete states of dose taking (1) and dose missing (0) was modeled in the logit domain and then transformed back to the probability space (between 0 and 1) as follows:

(6) $$P01=\left(\frac{e^{\Theta 1}}{1+e^{\Theta 1}}\right)$$

(7) $$P10=\left(\frac{e^{\Theta 2}}{1+e^{\Theta 2}}\right)$$

(8) P00=1–P01

(9) P11=1–P10

where P01, P10, P11, and P00 are transition probabilities between dose-taking and dose-missing states. When testing for covariate effects, covariates were linearly added in the logit domain of transition probability parameters.

Logistic and Markov models were developed without adding covariates into the model to compare between these two approaches. Based on the empirical Bayesian estimates of transition probabilities derived from these two approaches, adherence patterns were simulated using the ‘Markov chain’ package within the R software platform version 3.4.2 (R Core Team, 2017). The NTT was calculated from the simulated adherence patterns as an indicator of the cumulative index of non-adherence (Girard et al., 1998). Duration of action was assumed to be 24 h based on the nominal dosing frequency of daily dosing. The NTT of an individual was measured as the sum of all the days where dose was missed for two or more consecutive instances. Each individual transition matrix was used to simulate 100 Markov chains to account for stochastic noise. The NTT calculation was repeated for all 100 Markov chains, and the mean for each participant was used for plotting. The correlation between the observed NTT and the mean NTT predicted from 100 realizations of the Markov model was used as diagnostic plot for model development.

To evaluate the demographic and socio-behavioral data, a full covariate model approach was implemented (Gastonguay, 2011). First, all the covariates of interest were selected based on a prior statistical analysis from the Partners Demonstration Project using standard, multivariable generalized estimating equation modeling (Haberer et al., 2017a); all the significant covariates in that analysis were included in this analysis to form a full model. During subsequent steps, model reduction was carried out by dropping non-significant covariates from the model based on 95% confidence intervals (calculated from the standard error of the parameter estimate for each covariate). Any covariate that included unity in its confidence interval of the odds ratio (OR) was dropped from the model. With rest of the covariates, the model run process was continued until none of the covariates dropped out of the model. Covariates that had <5% of transitions in any one of the four transitions ([01], [11], [10] and [00]) were also dropped from the model. Data formatting and plotting was carried out using the software package R version 3.4.2 (R Core Team, 2017). Modeling of the data was performed using NONMEM^®^ (ICON, Ellicott City, Maryland, version 7.3) software package, with Intel/GFortran compilers with Perl-speaks-NONMEM as the interface (Parke et al., 1999; Lindbom et al., 2004). The Laplacian method was used for parameter estimation.

### Simulation of the Pharmacokinetic Profile

For visually illustrating the impact of covariate effects on PrEP adherence patterns and consequent pharmacokinetic (PK) profiles of TFV, a simulation exercise was performed. One typical subject was simulated for five of the significant covariates of the final model depending on the order of the size of their effect (higher ORs). The model estimated the typical probability values of each transition probability parameter (i.e., P01, P00, P10, and P11), which were used for simulating adherence patterns using the Markov chain package within R software (R Core Team, 2017). These adherence patterns were incorporated into a NONMEM data set to simulate a one-month dosing period. A population PK model of TFV reported by our group (Lu et al., 2017) in a subset of patients from this study was used to simulate the PK profiles. Comparative PK profiles for each of the significant covariates were plotted for each covariate.

## Results

### Demographic Data and Adherence Profiles

A total of 985 participants were enrolled into the study. Data of subjects with missing visits and missing data due to a broken or lost device were dropped from analysis. Data for 920 participants were available for inclusion into the analysis. The majority were men (n = 601, 65%), and ≥25 years of age (n = 737, 80%). Most of the participants (n = 900, 98%) started PrEP upon enrollment, with the exception of 16 (1.5%) subjects who started 1 month after enrollment and 4 (0.5%) who started more than 3 months after enrollment. A demographic summary of subjects is provided in **Table 1**, and a summary of adherence data is provided in **Supplementary Table 1**. The time course of adherence patterns in a few representative subjects is shown in **Supplementary Figure 1**.

**Table 1:** Demographic characteristics of study participants.

S No	Variables	Number (%)
	**Total**	**920 (100)**
**1**	Sex	
	Male	601 (65)
	Female	319 (35)
**2**	**Age**	
	Age >25 years	737 (80)
	Age <25 years	187 (20)
**3**	**Age difference**	
	Male partner > 5 years older than female	409 (44)
	Male partner NOT > 5 years older than female	511 (56)
**4**	**Sex risk**	
	No sex	40 (4)
	Sex with partner with 100% condom use	311 (34)
	Sex with partner less than 100% condom use	569 (62)
**5**	**ART status**	
	Partner on ART for 6 months	481 (52)
	Partner on ART for less than 6 months	439 (48)
**6**	**Concerns for taking PrEP**	
	No concerns	828 (90)
	Have concerns	92 (10)
**7**	**Relationship Desire**	
	Wants relation to succeed	809 (88)
	Not concerned with relation	111 (12)
**8**	**Pregnancy status and intentions**	
	Not pregnant and not trying	739 (80)
	Trying for pregnancy	63 (7)
	Pregnant	118 (13)
**9**	**Follow-up status**	
	More than 6 months follow-up	523 (57)
	Less than 6 months follow-up	397 (43)
**10**	**Relationship status**	
	Couple with study partner	914 (99)
	No longer couple	6 (1)
**11**	**Alcohol problem**	
	Problem alcohol use	184 (20)
	No alcohol problem	736 (80)
**12**	**PrEP initiation time**	
	PrEP started on enrollment	900 (98)
	PrEP started in 1 month	16 (1.5)
	PrEP started in 3+ months	4 (0.5)

All the demographic characteristics the data was captured at the base line. For time sensitive items like participant in the follow-up for 6 months, Partner in ART for 6 months, PrEP initiation time were captured at the end of follow-up period.

### Analysis of Transitions

A total of 258,714 transitions between dose-missing (0) and dose-taking (1) states were observed in the data. The transition [11] was the most commonly observed transition, accounting for 65% of the total transitions, whereas the transitions [01] and [10] were the least observed transitions, accounting for around 7.5%. Several covariates, i.e., concern for taking PrEP, continuing to be in a relationship with the study partner, and PrEP initiation time, had < 5% of the state transitions in some of their sub-categories. The summary of all four state transitions for all covariates is presented in **Supplementary Table 2**. A summary of transitions at an individual level are presented in **Supplementary Table 3**.

### Model Development

**Figure 2:**
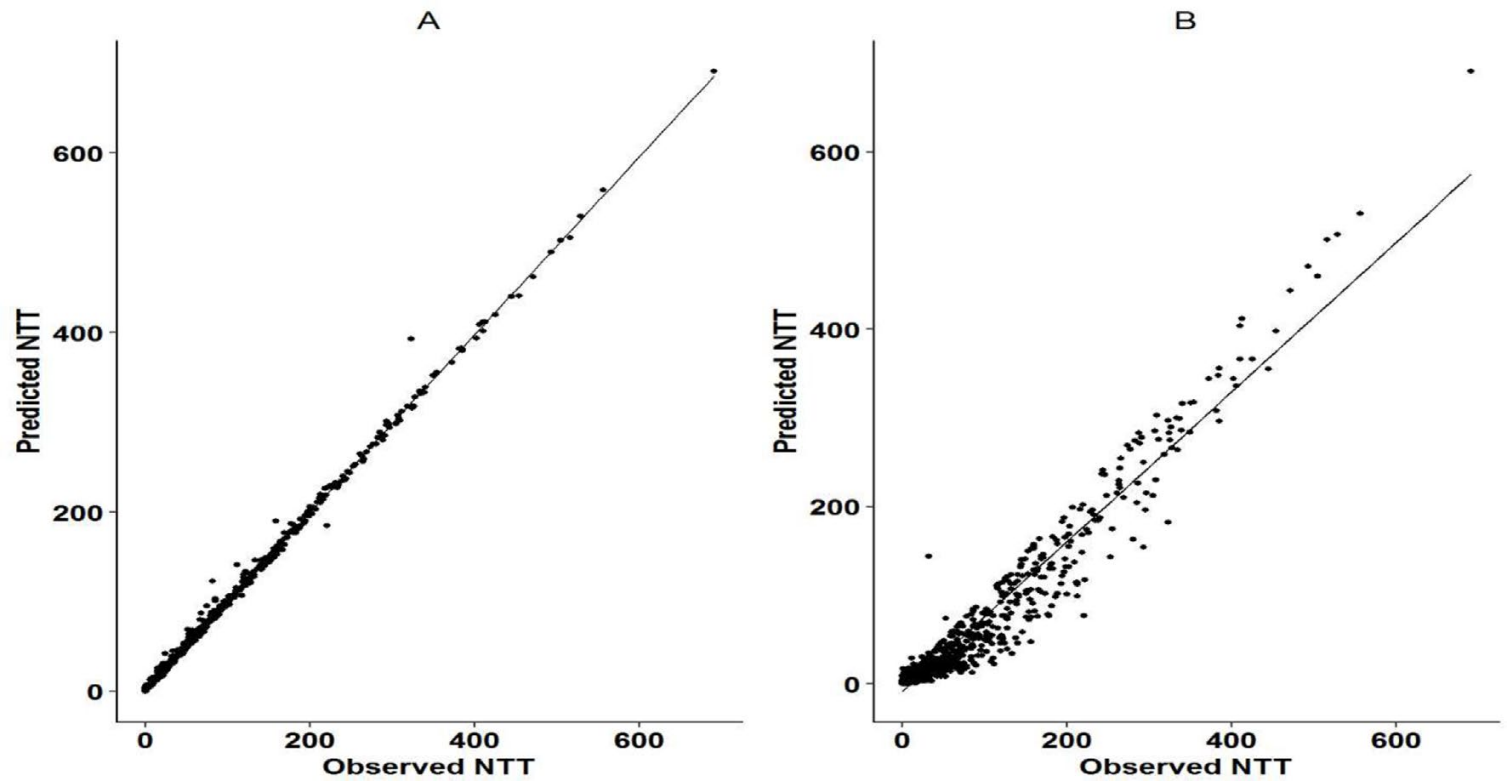
Observed vs predicted NTT for Markov model and logistic regression mode. Panel **A** shows the correlation between the observed and predicted NTT as per the Markov model. Panel **B** shows the correlation between the observed and predicted NTT as per the logistic regression model. NTT was calculated as the cumulative time (in days) that sequential doses were missed in a Markov chain. It was assumed that 24 h was the duration of action of each administered dose and two sequential missed doses were considered as one NTT. Cumulative sums were calculated for subsequent missing doses.

The Markov model resulted in a ∼50,000 point drop in the NONMEM objective function compared to the logistic model. The observed and the predicted numbers of transitions between dose-missing (0) and dose-taking (1) states are shown in **Table 2**. Individual probability parameters from the logistic and Markov models were used to generate the predicted NTT. The comparison between observed and predicted NTT between the logistic and Markov models is shown in **Figure 2**. This plot shows that the Markov model better predicts NTT than the logistic model. Thus, the Markov model was selected as the base model for further covariate analysis.

**Table 2:** Comparison of the predictability of state transitions in the adherence data between the logistic and Markov models: Base model development.

State Transition	Observed	Simulation 1	Simulation 2	Simulation 3	Simulation 4	Simulation 5
**Markov model predictions**						
[11]	**201119**	200090	199090	200327	200385	200241
[10]	**24431**	24652	24885	24725	24678	24515
[01]	**23986**	24392	24617	24459	24407	24237
[00]	**62747**	63149	63691	62772	62813	63290
**Logistic model predictions**						
[11]	**201119**	183053	183100	183203	183705	183190
[10]	**24431**	42070	42115	42099	42006	42043
[01]	**23986**	41809	41833	41831	41725	41735
[00]	**62747**	45351	45235	45150	44847	45315

Predicted transitions (counts) from both Markov and Logistic models. Four transitions ([11], [10], [01], [00]) of the predicted adherence data were compared against transitions in the observed data. A total of five simulations were carried out for both models to show the stochastic noise. Transitions predicted by the Markov model were closer to the observed values than the logistic model in all instances.

Seven significant covariates remained in the final model after the iterative model reduction process. The full list of covariates and results of iterative stages of model reduction steps are presented in **Supplementary Table 4**. The significant covariates were age, female sex, partner on ART for ≥6 months, desire for the relationship to succeed, study follow-up for ≥6 months, problem alcoholic use, and sex risk with partner with respect to condom use. When looking at the mechanisms of impact on adherence, the covariates acted in two different ways. First, certain covariates positively impacted adherence, when the participant re-initiated medication (transition [01]). Second, certain covariates negatively influenced adherence, when participants discontinued taking medications (transition [10]). For example, female participants and 25 years or older had lower odds of discontinuation. Those whose partners were on ART therapy for ≥6 months were more likely to discontinue PrEP and less likely to re-initiate.

Participants who desired the relationship with their partners to succeed were more likely to re-initiate PrEP and less likely to discontinue. Those who were in the follow-up for 6 months were more likely to discontinue PrEP and less likely to re-initiate.

Participants, who had an alcohol use problem, were more likely to discontinue PrEP and less likely to re-initiate. Those who reported sex with their partner and 100% condom use had lower odds of discontinuation, whereas those who reported less than 100% condom use had lower odds of discontinuation and higher odds of re-initiation of PrEP compared with those who reported no sex with their partners.

The impact of all significant covariates associated with the respective transition probability parameters is presented in **Table 3**. The mechanisms responsible for the impact of covariates on the adherence are presented in **Table 4**.

**Table 3:** Final model with significant covariates and their impact on adherence.

S. No	Covariates and impact on transitions	OR	95% CI	Interpretation
1.	Age >25 years on P10	0.61	0.49–0.77	Reduced P10 leading to increased adherence
2.	Female sex on P10	0.67	0.55–0.80	Reduced P10 leading to increased adherence
3.	Partner on ART for 6 months on P01	0.86	0.84–0.88	Reduced P01 leading to reduced adherence
4.	Partner on ART for 6 months on P10	1.34	1.30–1.38	Increased P10 leading to reduced adherence
5.	Wants relationship to succeed on P01	1.45	1.35–1.56	Increased P01 leading to increased adherence
6.	Wants relationship to succeed on P10	0.79	0.73–0.85	Reduced P10 leading to increased adherence
7.	In follow-up for 6 months on P01	0.80	0.79–0.82	Reduces P01 leading to reduced adherence
8.	In follow-up for 6 months on P10	1.25	1.22–1.28	Increased P10 leading to reduced adherence
9.	Problem alcohol use on P01	0.63	0.59–0.66	Reduced P01 leading to reduced adherence
10.	Problem alcohol use on P10	1.16	1.10–1.24	Increased P10 leading to reduced adherence
11.	Group with 100% condom use vs no sex group on P10	0.84	0.81–0.87	Reduced P10 leading to increased adherence
12.	Group with less than 100% condom use vs no sex group on P01	1.21	1.18–1.24	Increased P01 leading to increased adherence
13.	Group with less than 100% condom use vs no sex group on P10	0.69	0.67–0.72	Reduced P10 leading to increased adherence

The 95% confidence intervals were calculated with NONMEM asymptotic standard errors on covariate parameters using the following formula: CI = ^e^ *^θ^* ^+1.96(SE)^. Any covariate, which contained ‘1’ in its confidence interval was considered as non-significant and dropped from the model.

**Table 4:** Key mechanisms of covariates on PrEP adherence.

S.No.	Covariate	Impact on adherence	Key mechanism
1.	Age > 25 years	Increase	Reduced discontinuation
2.	Female sex	Increase	Reduced discontinuation
3.	Partner is on ART for 6 months	Reduce	Increased discontinuation and reduced re-initiation
4.	Wants relationship to succeed	Increase	Increased re-initiation and reduced discontinuation
5.	In the study for 6 months	Reduce	Increased discontinuation and reduced re-initiation
6.	Problem alcohol use	Reduce	Increased discontinuation and reduced re-initiation
7.	100% condom use in sex with partner compared to no sex group	Increase	Reduced discontinuation
8.	<100% condom use in sex with partner compared to no sex group	Increase	Increased re-initiation and reduced discontinuation

### Simulation of the PK profile

**Figure 3:**
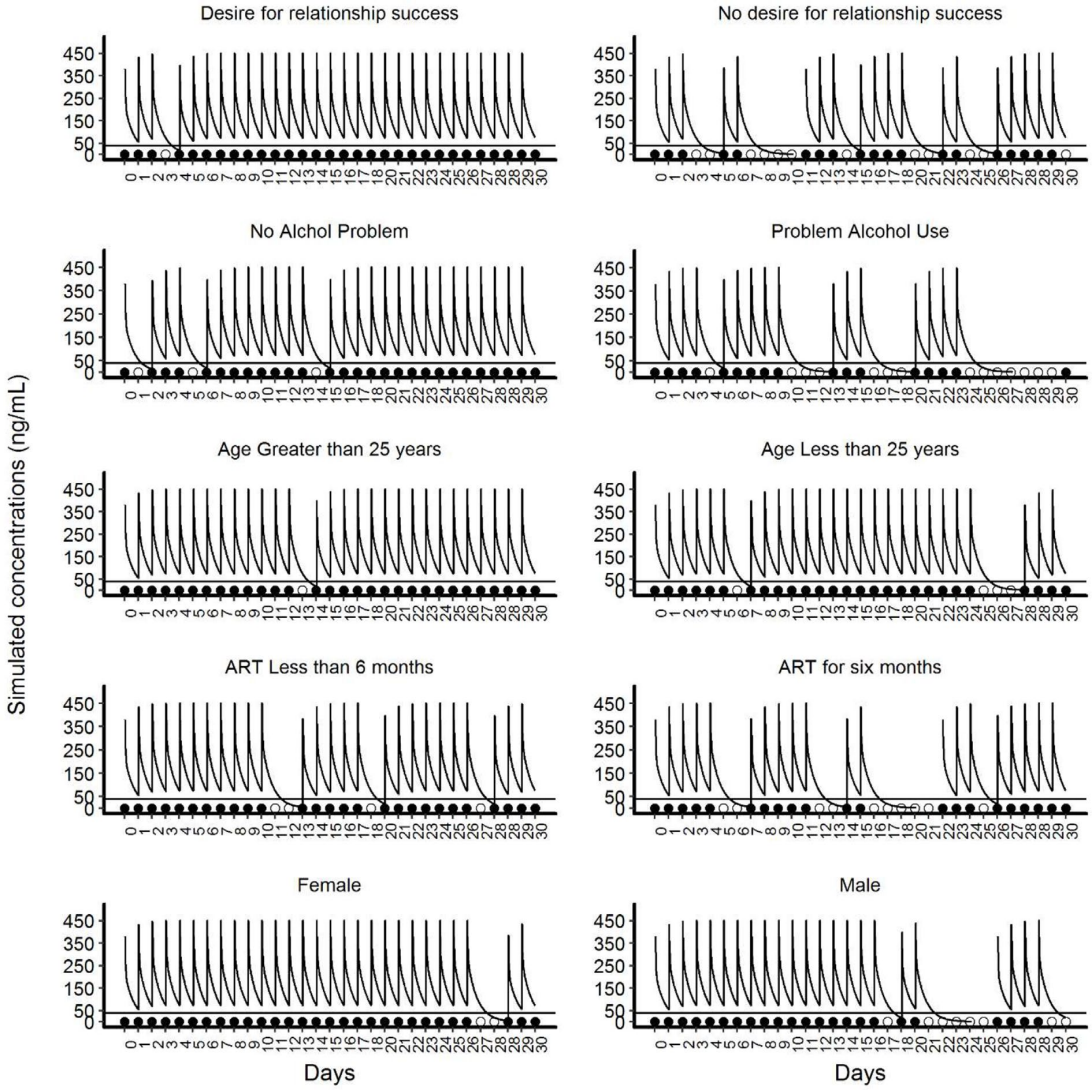
Illustrative simulated pharmacokinetic profiles for the categories of significant covariates of the final Markov model. This panel of plots presents illustrative simulated pharmacokinetic profiles based on the categories of five significant covariates. The panels on the left side show the categories of covariates that increase adherence and the panels on the right side show the categories of the covariates that reduce adherence. In each panel, the line that intercepts at 40ng/mL is the threshold level of protection for TFV. The filled circles near the x-axis represent “doses taken” and unfilled circles indicate ‘doses missed’.

The comparative PK profiles from the simulation are presented in **Figure 3**. These profiles illustrate the potential impact of non-persistence due to covariate effects on the average PK profile of TFV. For TFV, the estimated protective effect against HIV at plasma concentrations of >40/mL was 91% (Donnell et al., 2014). Non-persistence resulted in higher sequential drug omissions and caused the average PK concentrations to decrease below 40 ng/mL in participants with no desire for the relationship to succeed, with problem alcohol use, younger than 25 years, participants that with partners on ART for more than 6 months and male participants.

## Discussion

Our analysis identified several participant characteristics that are associated with non-adherence to PrEP in a Markov mixed-effects model framework. The covariate effects found to be significant are in general agreement with prior reports (Haberer et al 2013; Koenig et al., 2013; Amico and Stirratt, 2014; Corneli et al., 2014; Gengiah et al., 2014; Psaros et al., 2014; Kebaabetswe et al., 2015; Haberer et al., 2017a; Haberer et al., 2017b). The distinction of our approach was to utilize a parametric Markov model that provided insights into mechanisms of non-adherence (correlation to either P01 or P10) and predictive capabilities of adherence patterns in addition to the inferential analysis compared to the primary statistical analysis (Haberer et al, 2017a). The dependence of future state on the current state (persistence, execution) in the electronic adherence measurements was evident by the better fit of the Markov model compared with the logistic model. A similar observation was made in previous reports on the analysis of electronic adherence data (Girard et al., 1998; Madrasi et al., 2017). Some of the issues associated with ignoring the Markov element in the data when present include increased type I error rates on covariate inclusion, overestimation of information content in the data, and unrealistic simluation of individual time course of the outcomes (Silber et al., 2009).

In the present analysis, the Markov model predicted that NTT values were closer to the observed NTT in the data set than those of the logistic model, indicating a better description of adherence pattern. The logistic model predicted a much higher number of transitions between dose missing (0) and dose taking (1) compared with the Markov model and thus provides less predictive capability of adherence patterns. These findings support the use of the Markov modeling approach to describe adherence data compared to logistic modeling approach.

Age, sex, marriage or relationship status with partner, risk perception, concerns about taking PrEP, problematic alcohol use, age difference between partners, and sex with the partner or abstinence have been found to be important factors that influence adherence behaviors in other PrEP studies (Haberer et al 2013; Koenig et al., 2013; Amico and Stirratt, 2014; Corneli et al., 2014; Gengiah et al., 2014; Psaros et al., 2014; Kebaabetswe et al., 2015; Haberer et al., 2017a; Haberer et al., 2017b). In the present study, we found that many of these reported factors influencing PrEP adherence of study participants as a validation to the Markov modeling approach. Moreover, the assessment of transition states in the Markov model allow important insights into the mechanism by which these factors influence PrEP adherence.

For instance, participants older than 25 years and/or female sex had lower odds of transitioning from the dose-taking state to the dose-missing state and thus had higher persistence. Younger age has been reported to negatively influence PrEP adherence in other studies (Amico and Stirratt, 2014; Haberer et al., 2017a; Madrasi et al., 2017). This finding might be related to the level of maturity associated with age. The role of sex has been qualitatively explored in serodiscordant couples in Kenya. Carroll et al explored the sex power dynamics within households, which influenced adherence behaviors. They noted that in many instances, women were expected to be responsible for daily health care management tasks for them and their spouses, even though decisions were taken by their husbands. It was also noted that many seronegative men, but not women, found PrEP burdensome (Carroll et al., 2016).

Study participants who desired their relationship to succeed with their partners had higher odds of transitioning from dose-missing state to dose-taking state and lower odds of transitioning from dose-taking to dose-missing state compared with those without such desire. This covariate had the highest odds compared with other covariates in improving adherence. Married participants tend to have better PrEP adherence compared with those who were single (Amico and Stirratt, 2014; Corneli et al., 2014). The desire to continue a relationship with the partner could be a motivation for married subjects to continue PrEP. In the current study, couples had mutually disclosed their HIV serostatus; therefore, the uninfected partner had a more accurate perception of risk for HIV transmission, which may have resulted in better adherence.

Participants who were in the study for ≥ 6 months were more likely to transition from dose-taking state to dose-missing state and less likely to transition from dose-missing state to dose-taking state compared to those who were in the study for <6 months. This finding may reflect fatigue with taking PrEP for a relatively long period. Additionally, longer time of follow-up could reflect the bridge strategy for PrEP in this study (i.e., PrEP should be taken until the partner living with HIV had taken ART for >6 months). Indeed, those whose partners were on ART rfor >6 months had a tendency to miss medication. Risk perception by uninfected partners could have changed if they perceived their partners as being less infectious after six months on ART (assuming they were not concerned about the partner’s ART adherence and/or presence of outside sexual partnerships). It should be kept in mind, however, that duration of follow-up and partner time on ART were not necessarily equivalent, as many partners did not start ART until well into follow-up; these variables were thus tested independently in our model.

In the current analysis, it was observed that participants with problematic alcohol use were more likely to miss doses and continue in the same dose-missing state. Heavy use of alcohol has been similarly associated with poor adherence to PrEP (Amico and Stirratt, 2014; Haberer et al., 2017a).

Sex with the study partner or with others also influenced adherence to PrEP in this and other studies. Those who had sex with the study partner known to be living with HIV tended to have higher adherence compared to those who reported no sex with the study partner (Haberer et al., 2013; Kebaabetswe et al., 2015; Haberer et al., 2017a). In the current analysis, we observed that the participants who had sex with the partner, either with or without condoms, had higher adherence than those who reported no sex. These groups also had higher odds for persisting on PrEP; this finding might have been due to the higher level of risk perception by the uninfected partners of the serodiscordant couples. Those who abstained from sex with the study partner had lower adherence to PrEP possibly because of lower risk, although risk from any potential outside partnerships was unknown.

The current report agrees with our prior report (Madrasi et al., 2017) on Markov modeling of MEMS based adherence data on the approach and covariate effects. There are two important differences between these two analyses. First, the input data in the current analysis is from the Partners Demonstration Project (Haberer et al., 2017a) and the prior analysis used input data from the Partners PrEP Ancillary Adherence Sub-study (Haberer et al., 2013). Both studies evaluated a different set of covariates with common variables being age, sex, and problematic alcohol use. Thus, a direct comparison of results is not possible on all covariate effects. Second, a full covariate modeling approach was used in the current analysis compared to step-wise approach in the previous report. Findings on the common covariates between studies agreed well. Madrasi et al found that female sex and older age had a positive impact on adherence, which is similar to the current report. In addition, the current analysis identified problematic alcohol use as a significant covariate negatively associated with adherence, whereas it was not significant in prior report. The primary statistical analysis of both studies using regression modeling found similar results. The lack of significance in the Partners PrEP sub-study may reflect the lower prevalence of problematic alcohol use (10.6% versus 20% in current study).

The simulated PK profiles illustrated the impact of covariates on PrEP adherence and the subsequent effect on TFV levels. We do not imply a causative link of TFV levels to prophylactic efficacy. Rather, intracellular TFV-DP and FTC-TP levels are responsible for viral suppression and efficacy. Our Markov model can be linked to mechanistic HIV viral dynamic models (Duwal et al, 2016) to understand the onset and offset of prophylactic efficacy. The covariates that had negative impact on adherence may result in a PK profile with substantial length of time below the threshold value for protection. This situation may reduce PrEP effectiveness and may leave the participant unprotected in case of viral exposure during this period.

When interventions are planned to enhance adherence to therapy, it is important to understand associated issues and mechanisms of non-adherence. The Markov modeling approach identified significant covariates that impact adherence, along with mechanisms by which they act on adherence. Adherence interventions can be tailored based on the type and number of risky covariates in a subject. Intervention designs should consider the mechanisms of non-adherence, whether the subject has problems in initiation or with persistence to therapy.

There are several limitations to this study. Electronic adherence records may not be able to differentiate true dosing events and false-positive openings of MEMS containers, thus adding a certain level of uncertainty to the data. Unfortunately, the false-positive rate from the electronic adherence monitoring system cannot be known; however, it is generally low (Musinguzi et al., 2016). Additionally, some covariates used in the data reflect subjective information provided by participants, which cannot be verified independently. The results of the present study also have to be interpreted in the context of the socio-demographic and cultural background of the study participants. Finally, given the observational design of the study, there could be some unaccounted factors affecting adherence. Thus, the significant covariates identified in this analysis represent correlations rather than causality.

## Conclusions

The Markov mixed-effects modeling approach was used to study the impact of various factors on adherence to PrEP medications in serodiscordant couples. Female sex, older than 25 years, desire for the relationship with the partner to succeed, and use of condoms during sex with the partner were positively associated with PrEP adherence. Problematic alcohol use negatively associated with PrEP adherence. Although participation in the study for 6 months and the partner having taken ART for 6 months or more were found to be negatively associated with PrEP adherence, this finding is consistent with the bridge strategy and indicates potential for adherence to the bridge strategy to work well with this population. The developed Markov model provides insight into the stages of PrEP adherence (i.e., initiation, execution, and/or persistence) and can be used to develop further interventions to promote PrEP adherence.

## Partners Demonstration Project Team

Coordinating Center (University of Washington) and collaborating investigators (Harvard Medical School, Johns Hopkins University, Massachusetts General Hospital): Jared Baeten (protocol chair), Connie Celum (protocol co-chair), Renee Heffron (project director), Deborah Donnell (statistician), Ruanne Barnabas, Jessica Haberer, Harald Haugen, Craig Hendrix, Lara Kidoguchi, Mark Marzinke, Susan Morrison, Jennifer Morton, Norma Ware, Monique Wyatt.

## Project Sites

Kabwohe, Uganda (Kabwohe Clinical Research Centre): Stephen Asiimwe, Edna Tindimwebwa

Kampala, Uganda (Makerere University): Elly Katabira, Nulu Bulya.

Kisumu, Kenya (Kenya Medical Research Institute): Elizabeth Bukusi, Josephine Odoyo.

Thika, Kenya (Kenya Medical Research Institute, University of Washington): Nelly Rwamba Mugo, Kenneth Ngure.

Data management: DF/Net Research.

## Ethics Statement

The study protocol was approved by the University of Washington Human Subjects Division (STUDY00001674) and ethics review committees at each study site (Kabwohe: UNCST HS1410, NARC 135; Kampala: UNCST HS1289, NARC 126; Kisumu: KEMRI SSC NO 2441; Thika: KEMRI P286/05/2012). Participants provided written informed consent.

## Author Contributions

SM, AC, and JH developed the analysis. SM and AC conducted the modeling with significant input from MF, MS, CH, and JH. SM wrote the first draft of the paper, which was edited and approved by all authors.

## Funding

The Partners Demonstration Project was funded by the Bill and Melinda Gates Foundation (OPP1056051), the National Institute of Mental Health of the US National Institutes of Health (R01MH095507 and R01MH098744) and the United States Agency for International Development (AID-OAA-A-12-00023); the study also supported by the University of Washington/Fred Hutch Center for AIDS Research (P30 AI027757), supported by NIAID, NCI, NIMH, NIDA, NICHD, NHLBI, NIA, NIGMS, NIDDK of the National Institutes of Health. This work is made possible by the generous support of the American people through USAID; the contents are the responsibility of the authors and do not necessarily reflect the views of USAID, NIH, or the United States Government. PrEP medication was donated by Gilead Sciences.

## Conflict of Interest Statement

MF was employed by Trevena Inc, and MS was employed by Nuventra. JH has served as a consultant for Merck.

The remaining authors declare that the research was conducted in the absence of any commercial or financial relationships that could be construed as a potential conflict of interest.

The handling editor and reviewer MVK declared their involvement as co-editors in the Research Topic, and confirm the absence of any other collaboration.
