# Supplementary material for: Assessment of Demographic and Socio-Behavioral Factors on Adherence to HIV Pre-Exposure Prophylaxis Using a Markov Modeling Approach
Source: Front Pharmacol. 2019 Jul 12;10:785. doi: 10.3389/fphar.2019.00785 (PMC6639421; doi:10.3389/fphar.2019.00785)
Supplement: Supplementary file 1 [file DataSheet_1.docx]

**Supplementary Table 1. Data on dosing events, adherence and multiple opening records**

| **Category of Data** | **Counts and percentages** |
| --- | --- |
| **Dosing events** | |
| Total number of dosing events | 188016 |
| Mean doses per subject | 206 |
| Median number of doses [IQR] | 213 [88 – 320] |
|  | |
| **Adherence data** | |
| Mean adherence percentage | 71 |
| Median adherence percentage [IQR] | 81 [54 – 93] |
|  | |
| **Multiple MEMS cap opening events** | |
| Number of participants with multiple MEMS  opening records | 827 |
| Total multiple opening events | 9410 |
| Mean number of events per participant | 11 |
| Median number events [IQR] | 7 [4-14] |

**Supplementary Table 2. Number of transitions for covariates and their categories included in the full covariate model**

|  | **Transitions** | | | |
| --- | --- | --- | --- | --- |
|  | **[00]** | **[01]** | **[10]** | **[11]** |
| **Total number of transitions (%)** | 50619  (20) | 19592 (7.5) | 19986 (7.5) | 168517 (65) |
| **Covariates** |  |  |  |  |
| **Gender** Male | 35888 | 14304 | 14565 | 110248 |
| Female | 14731 | 5288 | 5421 | 58269 |
| **Age** Age >25 years | 39243 | 15450 | 15751 | 139839 |
| Age <25 years | 11376 | 4142 | 4235 | 28678 |
| **Age difference** |  |  |  |  |
| Male partner > 5 years older than  Female | 20974 | 8391 | 8558 | 78989 |
| Male partner NOT > 5 years older than  Female | 29645 | 11201 | 11428 | 89528 |
| **Sex Risk** |  |  |  |  |
| No sex | 8739 | 2630 | 2640 | 17321 |
| Sex with partner with 100% condom use | 27627 | 10048 | 10238 | 91171 |
| Sex with partner less than 100% condom use | 14253 | 6914 | 7108 | 60025 |
| **ART status** |  |  |  |  |
| Partner on ART for six months | 8224 | 3166 | 3195 | 24727 |
| Partner on ART for less than six months | 42395 | 16426 | 16791 | 143790 |
| **Concerns for taking PrEP** |  |  |  |  |
| No Concerns | 46347 | 18341 | 18699 | 160761 |
| Have Concerns | 4272 | 1251 | 1287 | 7756 |
| **Relationship desire** |  |  |  |  |
| Wants relation to succeed | 43784 | 17473 | 17801 | 153420 |
| Not concerned with relation | 6835 | 2119 | 2185 | 15097 |
| **Pregnancy status and intentions** |  |  |  |  |
| Not pregnant and not trying | 42233 | 15764 | 16059 | 137074 |
| Trying for pregnancy | 4097 | 1869 | 1910 | 15303 |
| Pregnant | 4289 | 1959 | 2017 | 16140 |
| **Follow-up status** |  |  |  |  |
| More than six months follow-up | 14514 | 5669 | 5708 | 41841 |
| Less than six months follow-up | 36105 | 13923 | 14278 | 126676 |
| **Relationship status** |  |  |  |  |
| Couple with study partner | 46900 | 18521 | 18916 | 163004 |
| No longer couple | 3719 | 1071 | 1070 | 5513 |
| **Alcohol problem** |  |  |  |  |
| Problem alcohol use | 11627 | 4578 | 4685 | 28456 |
| No alcohol problem | 38992 | 15014 | 15301 | 140061 |
| **PrEP initiation time** |  |  |  |  |
| PrEP started on enrollment | 48810 | 19366 | 19745 | 166849 |
| PrEP started in one month | 1436 | 219 | 232 | 1277 |
| PrEP started in three plus months | 373 | 7 | 9 | 391 |

**Supplementary Table 3. Data on transitions per participant**

| **Transition data** | **Transition 11** | **Transition 10** | **Transition 01** | **Transition 00** |
| --- | --- | --- | --- | --- |
| **Total number of transitions (258714)** | 168517 | 19986 | 19592 | 50619 |
| **Mean transitions per person** | 184 | 22 | 21 | 55 |
| **Median transitions per person [IQR]** | 177 [75-284] | 16 [6-32] | 15 [6-31] | 20 [3-70] |

**Supplementary Table 4 Markov model reduction steps from full covariate model**

| **Covariates tested**  **in full covariate model** | **Reduced Model -1** | **Reduced Model-2** | **Reduced Model-3** | **Final model** |
| --- | --- | --- | --- | --- |
|  | **Objective Function Value (OFV)** | | | |
|  | **172919.54** | **172927.35** | **173033.20** | **173033.28** |
|  | **Significant Covariates** | | | |
| AGE ON P01 & P10 | AGE ON P10 | AGE ON P10 | AGE ON P10 | AGE ON P10 |
| FEML ON P01 & P10 | FEML ON P10 | FEML ON P10 | FEML ON P10 | FEML ON P10 |
| ARTS ON P01 & P10 | ARTS ON P01 & P10 | ARTS ON P01 & P10 | ARTS ON P01 & P10 | ARTS ON P01 & P10 |
| NOCON ON P01 & P10 | NOCON ON P01 & P10 | NOCON ON P01 & P10 | - | - |
| RELAT ON P01 & P10 | RELAT ON P01 & P10 | RELAT ON P01 & P10 | RELAT ON P01 & P10 | RELAT ON P01 & P10 |
| SIXMO ON P01 & P10 | SIXMO ON P01 & P10 | SIXMO ON P01 & P10 | SIXMO ON P01 & P10 | SIXMO ON P01 & P10 |
| NOCOP ON P01 & P10 | NOCOP ON P01 & P10 | NOCOP ON P01 | - | - |
| ALCO ON P01 & P10 | ALCO ON P01 & P10 | ALCO ON P01 & P10 | ALCO ON P01 & P10 | ALCO ON P01 & P10 |
| RISK-FLAG 1 & 2 ON P01 & P10 | RISK-FLAG 1 ON P10 & 2 ON P01 & P10 | RISK-FLAG 1 ON P10 & 2 ON P01 & P10 | RISK-FLAG 1 ON P10 & 2 ON P01 & P10 | RISK-FLAG 1 ON P10 & 2 ON P01 & P10 |
| PRTRY FLAG 3&4 ON P01 & P10 | PRTRY FLAG 3 ON P01 | PRTRY FLAG 3 ON P01 | PRTRY FLAG 3 ON P01 | - |
| PRPIN FLAG 5&6 ON P01 & P10 | PRPIN FLAG 5 ON P10 | PRPIN FLAG 5 ON P10 | - | - |
| AGEDF ON P01 & P10 | - | **-** | **-** | **-** |
| **Abbreviations:** AGE – Age of the participant ; FEML – Female gender ; ARTS – Study partner is on Anti-retroviral therapy for six months ; NOCON – No concerns for taking PrEP regimen ; RELAT – Wants relationship to succeed ; SIXMO – On follow-up for six months ; NOCOP – No longer couples with study partner ; ALCO – Alcohol problem ; RISK FLAGS – risk categories based on sex with partner using/not using condoms ; PRTRY FLAGS – categories trying for pregnancy and already pregnant ; PRPIN – Categories of PrEP initiation at first and third months of enrolment ; AGEDF – Male study partner five years older than female partner ; P01 – Transition from dose missing to dose taking ; P10 – Transition from dose taking to dose missing | | | | |

**Supplementary Figure 1.**  **Time courses of observed typical adherence patterns**


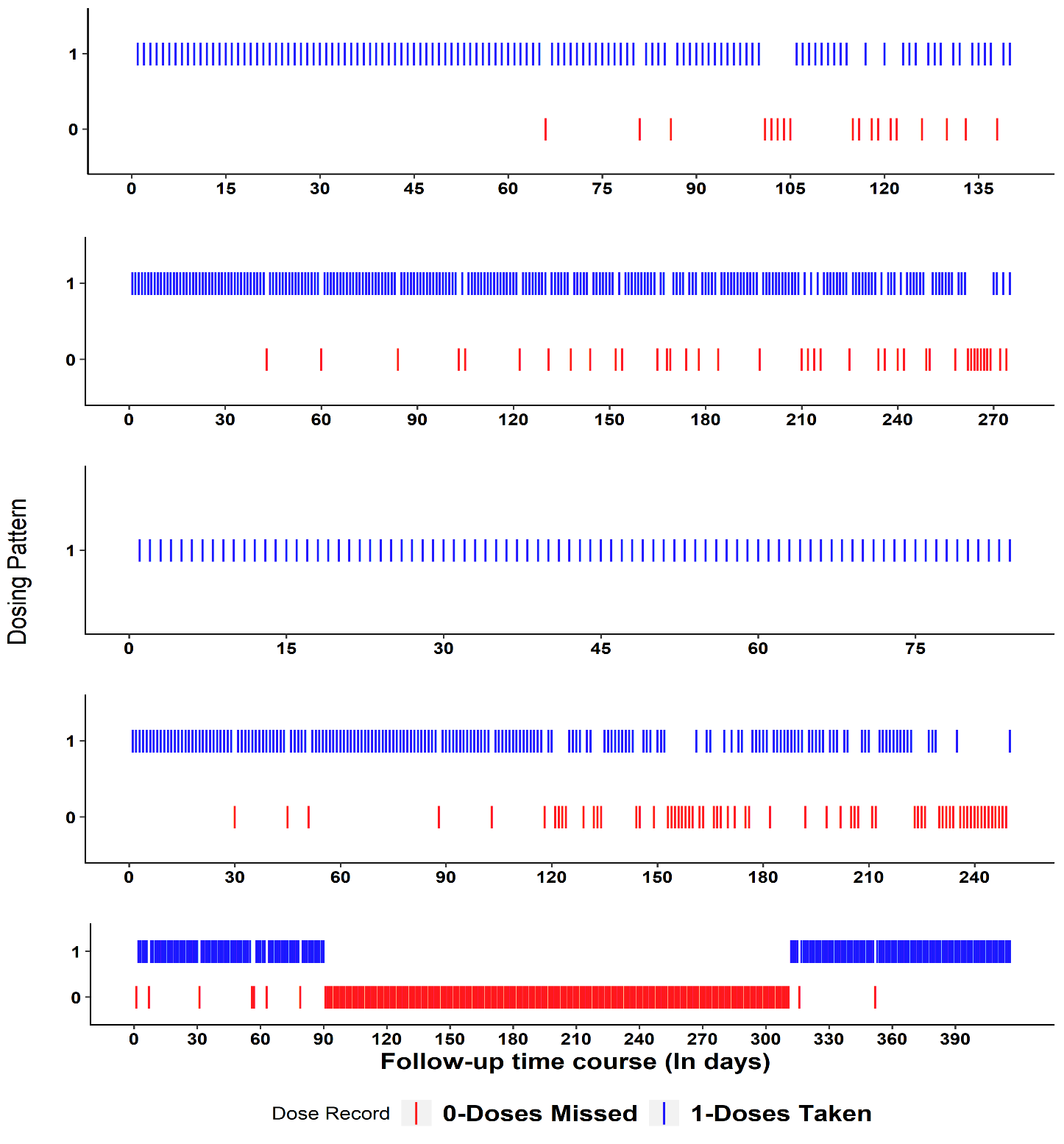


**Supplementary Text File 1**

**NONMEM control stream of the Markov Final model**

;-------------------------------------------------------------

;Markov Model

;920 subjects

;Full covariate model approach

;-------------------------------------------------------------

$SIZES NO=320000 LIM6=3000

$PROB MARKOV MODEL FOR MEMS DATA

$DATA MARKOV_MODEL_DATA_12_m.csv IGNORE = C

$INPUT C ID PTID VCODE DATE=DROP DV PDV PDVS FADH SADH AGE FEML AGEDF RISK ARTS NOCON RELAT PRTRY SIXMO NOCOP PRPIN ALCO

;PDV=Previous DV

;PDV = Value of immediately preceding observation

$PRED

FLAG1 = 0

FLAG2 = 0

FLAG3 = 0

FLAG4 = 0

FLAG5 = 0

FLAG6 = 0

IF(RISK.EQ.1)THEN

FLAG1 = 1

FLAG2 = 0

ELSEIF(RISK.EQ.2)THEN

FLAG1 = 0

FLAG2 = 1

ELSEIF(RISK.EQ.0)THEN

FLAG1 = 0

FLAG2 = 0

ENDIF

IF(PRTRY.EQ.2)THEN

FLAG3 = 1

FLAG4 = 0

ELSEIF(PRTRY.EQ.3)THEN

FLAG3 = 0

FLAG4 = 1

ELSEIF(PRTRY.EQ.1)THEN

FLAG3 = 0

FLAG4 = 0

ENDIF

IF(PRPIN.EQ.1)THEN

FLAG5= 1

FLAG6 = 0

ELSEIF(PRPIN.EQ.2)THEN

FLAG5= 0

FLAG6 = 1

ELSEIF(PRPIN.EQ.0)THEN

FLAG5 = 0

FLAG6 = 0

ENDIF

LOGIT1=THETA(1)+(THETA(3)*AGE)+(THETA(5)*FEML)+(THETA(7)*ARTS)+(THETA(9)*NOCON)+(THETA(11)*RELAT)+(THETA(13)*SIXMO)+(THETA(15)*NOCOP)+(THETA(17)*ALCO)+(THETA(19)*FLAG1)+(THETA(21)*FLAG2)+(THETA(23)*FLAG3)+(THETA(25)*FLAG4)+(THETA(27)*FLAG5)+(THETA(29)*FLAG6)+(THETA(31)*AGEDF)+ETA(1)

LOGIT2=THETA(2)+(THETA(4)*AGE)+(THETA(6)*FEML)+(THETA(8)*ARTS)+(THETA(10)*NOCON)+(THETA(12)*RELAT)+(THETA(14)*SIXMO)+(THETA(16)*NOCOP)+(THETA(18)*ALCO)+(THETA(20)*FLAG1)+(THETA(22)*FLAG2)+(THETA(24)*FLAG3)+(THETA(26)*FLAG4)+(THETA(28)*FLAG5)+(THETA(30)*FLAG6)+(THETA(32)*AGEDF)+ETA(2)

; convert back to probabilities

PV01 = EXP(LOGIT1)/(1+EXP(LOGIT1))

PV10 = EXP(LOGIT2)/(1+EXP(LOGIT2))

; CALCULATE STATE PROBAILITIES

; PR01 IS PROBABILITY OF MISSING LAST AND TAKING THIS DOSE

; PR00 IS PROBABILITY OF MISSING LAST AND MISSING THIS DOSE

; PR10 IS PROBABILITY OF TAKING LAST AND MISSING THIS DOSE

; PR00 IS PROBABILITY OF TAKING LAST AND TAKING THIS DOSE

PR01 = PV01

PR00 = 1-PV01

PR10 = PV10

PR11 = 1-PV10

IF(PDV.EQ.0.AND.DV.EQ.1) Y=PR01

IF(PDV.EQ.0.AND.DV.EQ.0) Y=PR00

IF(PDV.EQ.1.AND.DV.EQ.0) Y=PR10

IF(PDV.EQ.1.AND.DV.EQ.1) Y=PR11

$THETA (-0.36);#1 PROB of dose taking given dose missed

$THETA (-1.35);#2 PROB of dose missing given dose taken

$THETA (0 FIX);#3 EFFECT of AGE ON PV01

$THETA (-0.49);#4 EFFECT of AGE ON PV10

$THETA (0 FIX);#5 EFFECT of FEML ON PV01

$THETA (-0.41);#6 EFFECT of FEML ON PV10

$THETA (-0.15);#7 EFFECT of ARTS ON PV01

$THETA (0.29) ;#8 EFFECT of ARTS ON PV10

$THETA (0 FIX);#9 EFFECT of NOCON ON PV01

$THETA (0 FIX);#10 EFFECT of NOCON ON PV10

$THETA (0.37) ;#11 EFFECT of RELAT ON PV01

$THETA (-0.23);#12 EFFECT of RELAT ON PV10

$THETA (-0.22);#13 EFFECT of SIXMO ON PV01

$THETA (0.22) ;#14 EFFECT of SIXMO ON PV10

$THETA (0 FIX);#15 EFFECT of NOCOP ON PV01

$THETA (0 FIX);#16 EFFECT of NOCOP ON PV10

$THETA (-0.47);#17 EFFECT of ALCO ON PV01

$THETA (0.15) ;#18 EFFECT of ALCO ON PV10

$THETA (0 FIX);#19 EFFECT of RISK-FLAG1 ON PV01

$THETA (-0.18);#20 EFFECT of RISK-FLAG1 ON PV10

$THETA (0.19) ;#21 EFFECT of RISK-FLAG2 ON PV01

$THETA (-0.37);#22 EFFECT of RISK-FLAG2 ON PV10

$THETA (0 FIX);#23 EFFECT of PRTRY-FLAG3 ON PV01

$THETA (0 FIX);#24 EFFECT of PRTRY-FLAG3 ON PV10

$THETA (0 FIX);#25 EFFECT of PRTRY-FLAG4 ON PV01

$THETA (0 FIX);#26 EFFECT of PRTRY-FLAG4 ON PV10

$THETA (0 FIX);#27 EFFECT of PRPIN-FLAG5 ON PV01

$THETA (0 FIX);#28 EFFECT of PRPIN-FLAG5 ON PV10

$THETA (0 FIX);#29 EFFECT of PRPIN-FLAG6 ON PV01

$THETA (0 FIX);#30 EFFECT of PRPIN-FLAG6 ON PV10

$THETA (0 FIX);#31 EFFECT of AGEDF ON PV01

$THETA (0 FIX);#32 EFFECT of AGEDF ON PV10

$OMEGA

3.64

1.72

;$COV COMPRESS PRINT=E

;$SIGMA 0 FIX

$ESTIMATION NUMERICAL SLOW METHOD=COND LAPLACE LIKE MAXEVAL=9999 PRINT=5 MSFO = MSF31

$TABLE ID DV PDV PR00 PR01 PR10 PR11 NOPRINT ONEHEADER NOAPPEND FILE=results.txt
